# Designing, facilitating, and scaling-up video-based professional development: supporting complex forms of teaching in science and mathematics

**DOI:** 10.1186/s40594-017-0087-y

**Published:** 2017-11-20

**Authors:** Miray Tekkumru-Kisa, Mary Kay Stein

**Affiliations:** 1 Learning Systems Institute & School of Teacher Education, Florida State University, Tallahassee, FL USA; 2 University of Pittsburgh, Learning Research and Development Center, Pittsburgh, PA USA

**Keywords:** Video, Professional development, Teacher learning, Teacher leader, Professional development facilitator

## Abstract

This special issue brings together four effective video-based professional development programs—two in mathematics and two in science—that use classroom videos as the centerpiece of their efforts and have “scaling up” as their goal. In each paper, the authors surface their design considerations for creating scalable and sustainable video-based professional development interventions, including the challenges and successes they have experienced. The papers also emphasize the role of professional development facilitators in the scalability and sustainability of their programs and how best to support this emerging class of education professionals. This introductory paper situates these papers within the current literature on the design and facilitation of video-based professional development programs.

In the United States (US) and globally, attention is on the teaching and learning of Science, Technology, Engineering, and Mathematics, commonly referred to as STEM. Alongside calls for higher levels of college and career readiness and workforce preparation, policymakers and practitioners are recognizing the need to seriously upgrade instructional practices in all of the STEM subjects, but especially in science and mathematics. In the US, the Common Core State Standards for Mathematics and the Next Generation Science Standards have begun to provide a vision for what high-quality instruction in mathematics and science should look like, a vision that—unfortunately—departs radically from common practice (Banilower et al., 2013; Marrongelle et al., 2013; National Academies of Sciences, Engineering, and Medicine, 2015).

The transformation that needs to occur for improving instructional quality in science and mathematics classrooms demands a sustained focus on the professional development (PD) of science and mathematics teachers because, undoubtedly, teachers will play a central role in ensuring that these transformations happen and that they result in students’ readiness for college or the workplace. As stated in the recent report by the National Academies of Sciences, Engineering, and Medicine (2015) on science teachers’ learning:

Individuals increasingly must understand science and technology to thrive in today’s society, and schools accordingly are challenged to provide high-quality science learning experiences to all students. Teachers are at the forefront of meeting this challenge, and the quality of their instruction therefore acts as a major fulcrum for improving science education (p. 11).

In recent years, this recognition of the role of PD in transforming the quality of teaching has led to the development of new programs. This special issue presents four papers, each focusing on a different PD program but all of which grapple with questions regarding how to better support teachers’ learning to help them enact the vision called for by recent reforms. The four programs include two in science (Teaching Science with Cognitive Demand-PD [TSCD] by Tekkumru-Kisa and Stein and Science Teachers Learning from Lesson Analysis [STeLLA] by Roth and colleagues) and two in mathematics (Learning and Teaching Geometry [LTG] by Jacobs, Seago, and Koellner and the Problem-Solving Cycle [PSC] by Borko, Carlson, and colleagues). Common across these projects is their careful, theory-informed design of teacher professional development and their commitment to supporting the spread and sustainability of their programs. In the following section, we review the overarching ideas that drive this special issue and our reasons for including the PD programs noted above. We then turn to the projects themselves and preview some of the successes and challenges that they encountered.

## An introduction to the overarching ideas

### Designing for teacher learning

The projects described herein encompass innovative approaches to PD, approaches that built on contemporary ideas about the nature of cognition, learning, and teaching (Greeno et al., 1996). They also represent a shift from using workshops to teach techniques to using different PD strategies to build teachers’ understanding of subject matter, pedagogy, and student thinking (Stein et al., 1999). Following situative learning theory (Greeno, 2006), the designers have located teachers’ learning opportunities in their classroom activities (Ball & Cohen, 1999; Borko & Koellner, 2008; Putnam & Borko, 2000). As such, their work builds on a growing consensus that teachers’ learning should be situated within the artifacts of instructional practice (Ball & Cohen, 1999; Borko et al., 2008). For example, Reiser (2013) suggested for the PD to support NGSS vision to include rich images of classroom enactment to facilitate teachers’ sense making.

Prior research has identified features associated with PD that can support teachers as they transition to adopting ambitious instructional practices (Garet et al., 2001; Hawley and Valli, 1999; Loucks-Horsley et al., 2010; Penuel et al., 2007). Many features identified as successful are “practice-based” meaning that they are close to teachers’ day-to-day practice and involve the use of classroom artifacts and/or different representations of instruction (Ball & Cohen, 1999; Putnam & Borko, 2000). One feature that shows exceptional promise is the use of videos of teachers’ own and others’ instructional practice (Borko et al., 2008; Brophy, 2004; Sherin 2004).

As a particular manifestation of practice-based professional development, videos focus participants’ thinking and talk on artifacts from real classrooms and capture teaching in all of its complexity while, at the same time, affording space and time for guided reflection (Miller & Zhou, 2007; Sherin, 2004). Based on Gaudin and Chaliès’ (2015) recent review of international literature about the use of video in teacher education and PD, video viewing has become a prominent part of PD of teachers in all subject areas, at all grade levels, and on nearly every continent. There has been growing evidence about the positive effects of video-based PD programs on teachers’ learning and instructional practices e.g., (Tekkumru-Kisa & Stein, 2015; Borko et al., 2011; Koellner & Jacobs, 2015; Roth et al., 2011; Sherin & Han, 2004; van Es & Sherin, 2010) leading to a growing number of high-quality video-based PD programs for science and math teachers.

Although successful video-based PD programs have been developed, the next—even larger—challenge is to devise strategies for taking effective video-based PD programs beyond pockets of excellence to a larger scale. Developing sustainable and scalable PD programs is important to meet an increasing demand for teacher learning opportunities (Borko et al., 2014; Marrongelle et al., 2013; Wilson, 2013). This special issue brings together four effective video-based PD programs that use classroom videos as the centerpiece of their efforts and have “scaling up” as their goal. In each paper, the authors surface their *design considerations* for creating scalable and sustainable video-based PD interventions and the challenges and successes they experienced as they tried to scale-up their PD interventions.

### A missing piece of the puzzle: PD facilitators

A deep understanding of what PD should look like is only part of the equation (Borko et al., 2014). It is now widely recognized that the well-prepared facilitator is an essential component of the equation for ensuring PD’s effectiveness (Borko et al., 2014; Elliott et al., 2009; Koellner et al., 2011). Discussing high-quality PD without focusing on the facilitators and their role is like discussing high-quality instruction without mentioning teachers and their role. The evaluations of National Science Foundation’s Local Systemic Change Initiative Projects found that a high-quality leader makes a difference in the effectiveness of PD programs to support teachers’ learning (Banilower et al., 2006). Similarly, Clark et al. (2008) attributed the emergence of mathematically rich discourse within a teacher professional learning community in the PD project they examined to the active role taken by the designated facilitator in modeling productive discourse among the teachers. They went on to suggest that highly skilled facilitation requires specific training and coaching of the facilitator. In fact, some researchers (e.g., Borko et al., 2011; Borko et al., 2014; Coles, 2013; Elliott et al., 2009) have begun to focus on the development of PD facilitators in order to move their programs to different contexts and ensure the sustainability of these programs (Gaudin and Chaliès, 2015).

The importance of the role of the facilitator is also emphasized in reports on the implementation of instructional reforms. For example, one of the recommendations presented in a recent report by the National Academies of Sciences, Engineering, and Medicine (2015) about professional supports for practicing science teachers was the need for more research and development efforts to support PD facilitators. It states, “… Also lacking in the research literature are studies of how teachers learn to become leaders, as well as research that examines the role, expertise, or preparation of science professional development providers and facilitators (p. 228).” Similarly, in mathematics, one of the recommendations for designing and sustaining high-quality professional development systems in the Common Core State Standards era was the preparation and use of knowledgeable facilitators (Marrongelle et al., 2013). It stated:

Teachers and administrators play an important role in delivering professional development at scale and we must better understand how they are prepared to facilitate and support the implementation of school-based professional development. There is an emerging body of research on mathematics leaders that begins to identify how to cultivate teacher-leaders to support instruction aligned with the CCSSM mathematical practices (e.g., Elliott et al., 2009). We must continue such lines of research and further these lines of inquiry to better understand what works, for whom, and under what conditions (p. 208).

All of these suggest that, despite a consensus on the important role played by PD facilitators, researchers are just beginning to characterize what they need and how to support them (e.g., Elliott et al., 2009; Koellner et al., 2011; Jackson et al., 2015; Lesseig et al., 2016; Schifter & Lester, 2005). This special issue is one attempt to address this challenge by bringing together a group of researchers, whose work primarily focuses on PD facilitation, to uncover the ways in which they train PD facilitators to deliver their video-based PD programs. This is important because, as emphasized by others (e.g., Gaudin and Chaliès, 2015; Goldsmith & Seago, 2011; van Es et al., 2014; van Es et al., 2015), teachers do not learn by simply watching videos of classroom instruction; they learn from watching, discussing, and analyzing videos under the skillful guidance of a facilitator as part of a carefully designed, theoretically informed professional development.

### A closer look into the papers: each representing a particular phase of PD research

Borko (2004) organized programs of research on PD into three phases, each building on the previous one: Phase 1 research activities focus on an individual PD program at a single site; researchers provide initial evidence that the program can have a positive impact on teacher learning. Phase 2 research activities focus on a single PD program enacted by more than one facilitator at more than one site; in this phase, researchers are interested in the fidelity and thus examine whether the program can be enacted with integrity in different contexts and by different facilitators. Phase 3 research activities compare multiple PD programs, each enacted at multiple sites, and investigate their impact on teacher and student learning.

At the time of Borko’s publication, the vast majority of the PD research could be categorized into phase 1; research on phase 3 was scarce. Since that time, several phase 2 (e.g., Bell et al., 2010) and phase 3 (e.g., Heller et al., 2012; Penuel et al., 2011) types of research studies have been conducted (Borko et al., 2014).

For this special issue, we identified four PD projects, each of which represents a particular phase of PD research as described by Borko (2004). Tekkumru-Kisa and Stein focus on an individual video-based PD program at a single site (phase 1); Borko and colleagues as well as Jacobs, Seago, and Koellner focus on a single video-based PD program enacted by different facilitators in different sites (phase 2). Finally, Roth and colleagues focus on a video-based PD program enacted at multiple sites (phase 3). We purposefully sampled in this way in order to surface the growing demands placed on PD designers as they progress through these phases. For example, unlike phase 1 designers, phase 2 designers must take on the preparation and use of knowledgeable facilitators. They also have to design for issues of fidelity and adaptation that will inevitably arise in new sites. How much flexibility should be designed into the program? What parts are adaptable; what parts are not? Who should make adaptations? When?

Phase 3 designers need to deal with all of the above issues *plus* do so at multiple sites and assemble a research infrastructure that is up to the task of comparing their program with other PD programs. By surveying PD programs at each of these phases, we are positioned to learn how designers’ foci, tools, and resources evolve as they transition from a single program to one that is scalable beyond the initial developers’ vision. By limiting our sample to video-based PD projects, we are positioned to learn about the particular challenges and affordances of video at each of these phases.

Tekkumru-Kisa and Stein propose a framework for designing and facilitating video-based PD and also describe the design and facilitation of the TSCD video-based PD through the lens of this framework. TSCD-PD has been implemented one time and thus would be considered as an example of phase 1 PD research. Borko stated:

The goal of Phase 1 activities is to create existence proof … The resulting existence proofs unquestionably are an important contribution to the field. As Shulman (1983) reminded us, they “evoke images of the possible … not only documenting that it can be done, but also laying out at least one detailed example of how it was organized, developed, and pursued” (p. 495)

In phase 1, the facilitator and context of the PD remain unstudied. Even though Tekkumru-Kisa and Stein analyzed facilitation moves, their focus was on the features of the PD program (not the facilitator). Their goal was to articulate the design features of TSCD-PD (including features related to video-based discussions) so that TSCD-PD could be implemented and maintain its integrity when moved to phase 2.

Phase 2 research studies aim to determine whether the PD program can be enacted with fidelity in different settings and by different PD facilitators (Borko, 2004). Jacobs and colleagues focused on the fidelity of implementation of the LTG-PD by analyzing the preparation process of a PD facilitator, her use of PD materials and her rationale for the adaptations she made. Borko and colleagues report on the implementation of PSC model of PD and Mathematics Leadership Preparation model of PD leader preparation (Borko et al., 2015) within an urban school district through a Design-Based Implementation Research project. Even though context remains unstudied in phase 2 research, given the collaborative design effort with the district leaders within a DBIR research project, context of the school district becomes an important factor in their paper. The authors emphasize for the PD researchers the importance of being responsive to the context of the school partners if they expect their work to be meaningful.

Finally, phase 3 research studies are about comparing multiple PD programs, each enacted at multiple sites. In phase 3 research, the relationship among the facilitator, PD program, teachers, and context is studied (Borko, 2004). Roth and colleagues focus on the STeLLA video-based PD program. As such, they do not technically compare multiple PD programs. They are, however, one of a very few PD programs in science that has scaled to multiple contexts and has been implemented by different facilitators. Additionally, they have tested variations of the STeLLA PD program including one that was of equal duration but focused on content deepening and did not include any video analysis work.

## Previewing the papers: consistent themes presented for further thought

Looking across the four papers, common themes emerge regarding how the projects responded to challenges associated with their goal of creating a rigorous program that could be both scalable and sustainable. Concrete examples of authors struggling with these issues are provided below, along with a brief overview of the ways in which the authors reached across educational “layers” to inform their design work.

### Opening the black box: how were the PD programs designed and facilitated

In an NCTM-commissioned study of the field’s readiness for the CCSS, Marrongelle et al. (2013) identified the need for more studies that “open the black box of professional development” (p. 209). Given the increasing interest in and demand for development of video-based PD programs, we believe that clearly articulating the design features of effective PD programs and revealing the intentions and rationales of PD designers through a rich description of their programs will help the field to think more carefully about the contents of the black box. Importantly, the need for carefully articulated design principles does not end with specification of the program itself but extends to principles that guide how to set up and orchestrate the interactive discussions that occur as the program is enacted. Borko et al. (2014) argued for articulating *the* ***practice*** *of leading high-quality PD* to be able to define how these programs contribute to changes in teachers’ learning, instructional practices, and students’ learning.

Across these four projects, we expose the thinking and decision-making that occurred behind the scenes in facilitators’/designers’ minds as they planned and led video-based PD. In the course of describing their most recent research and design efforts, the paper authors (who are also the designers of the PD) make transparent the design decisions involved in setting up and orchestrating the programs, including the role of different frameworks and tools in their behind-the-scenes work.

For example, the essential design consideration underlying Jacobs and colleagues’ LTG-PD program was its high specification and expectation of fidelity and/or reasonable adaptations defined in terms of when and how to adapt. A second design consideration was the concept of “video in the middle,” an underlying structure of pre- and post-activities wrapped around a video as a way to get the most out of each video viewing event. In contrast to Jacobs and colleagues, Borko et al. characterized PSC-PD as a highly adaptable model, which—from the beginning—was designed to allow for tailoring it to the goals, interests, and needs of participating districts. Each of these decisions will have far-reaching impact on the shape and form of future iterations of these two PD programs; exposing the decisions around fidelity/adaptation marks the decision point for novice designers who otherwise might be surprised with “lethal mutations” (Brown, 1992) as they try to expand their programs to new sites.

These and other design decisions are commonly hidden from view: They are shared assumptions among the “founding” designers but left unarticulated to others. These assumptions undergird a wide range of decision-making-in-the-moment for this initial group. Without exposing these tacit beliefs/assumptions, however, others new to PD facilitation/design will flounder. One of the critical design considerations in the Tekkumru-Kisa and Stein program was “an approach to learning that first set up learner-led exploratory experiences, followed by experiences that channeled that exploration a bit, to a culminating experience in which the learning is directly addressed by the teacher/facilitator and connected to learners’ initial less-structured experiences” (p. 38). Knowing and understanding this structure could be immensely helpful to a novice facilitator. In the Roth et al. paper, the most mature of the PD efforts examined, the majority of the material focuses on elucidating the design principles associated with STeLLA, its *implementation* and its *scaling up*. The authors welcomed the opportunity to write about these undergirding—but mostly unspoken—aspects of their work. The STeLLA design principles are made even more vivid by their contrast with traditional research findings on what constitutes effective PD.

Exposing design decisions goes beyond decisions about the PD and teachers. This is especially evident in the case of video-based PD. In several of the studies, video was used not only for supporting teachers’ learning but also for supporting facilitators’/teacher leaders’ learning to orchestrate PD discussions. Therefore, while the papers discuss how they capitalized on the power of video in their projects, several of them also elaborated on how they supported facilitators’ learning to use video to effectively support teachers’ learning through videos of others leading similar PD discussions.

### Preparing PD leaders for scalable and sustainable PD programs

As noted earlier, there is a growing demand for widespread and high-quality professional development across the nation. However, there is a lack of systematic research on how best to train facilitators to both deliver well-designed PD with fidelity when appropriate and to learn when and how to best adapt the program (Borko et al., 2011, 2014; Elliott et al., 2009; Lesseig et al. 2016). Even’s international review of the literature on the facilitation of PD programs focused on this missing piece in the literature (Even, 2008). She points out the ill-defined nature of the field and lack of systematic investigations of PD facilitators, which may present challenges for designers of tools and programs to support PD facilitators.

Over the past decade, however, some research has begun to emerge that focuses on developing PD leaders’ capacity to design and facilitate high-quality PD to support high-quality instructional practices and improve student gains (e.g., Borko et al. 2014; Elliott et al., 2009; Jackson et al., 2015). Borko et al. (2014) stated “A central component of a sustainable, scalable PD model is the ability to prepare PD leaders who can adapt the model to a variety of local contexts and advocate for school and district support of the PD while maintaining integrity to its goals and design” (p. 151). Koellner et al. (2011), for instance, worked with novice teacher leaders to help them incorporate important features of PD programs including (1) fostering a professional learning community, (2) developing teachers’ mathematical knowledge for teaching, and (3) adapting PD to support local needs and interests, as they learn to facilitate Problem-Solving Cycle in their own schools. In a related study, Lesseig et al. (2016) studied how their use of video cases supported leaders’ noticing of the work required to facilitate a mathematics PD.

Some recent studies have also begun to articulate specific knowledge and skills leaders need to facilitate teachers’ learning including deep subject matter knowledge, familiarity with the special affordances and challenges of video, and appreciation of the beliefs and understandings of session participants (Lesseig et al., 2016; Coles, 2013; Schifter & Lester, 2005). Considering all these potential challenges and the needs of PD facilitators, the question turns to how novice facilitators’ skills and knowledge can be developed and by whom.

The PD programs represented in this issue build on this small but growing literature to take on this question. All of the papers address how to support the work of PD leaders and emphasize the role they play for the scalability and sustainability of the programs. In the paper by Jacobs and colleagues, we learn about the kinds of support provided to their facilitator-in-training (Hannah) during conference calls scheduled before and immediately after PD sessions as well as providing the opportunity for Hannah to rehearse some of the sessions. The rehearsal, in particular, is important for marking the idea that the PD design cannot be captured solely in materials but that it also consists of interactions between participants and the leader that support a particular kind of learning environment. An important way in which Roth et al. support facilitators-in-training is through detailed PD leader guides that include specific teacher learning goals for each PD session (building off of their knowledge that teachers implement more coherent lessons when they are guided by explicit student learning goals (see more below for building off of classroom research)).

In the paper by Borko and colleagues, authors provide insights into the adaptations they needed to make in their PD program as they worked with an urban district to develop their capacity to support mathematics reform, the most notable of which pertained to teacher leaders’ support. Because the novice leaders were less steeped in the curriculum than expected, the designers strengthened their modeling and debriefing activities with them as well as provided extended support to help them learn to select video clips that would be discussed in the PD sessions. Finally, Tekkumru-Kisa and Stein (in anticipation of training future facilitators in their PD program) propose a framework that can be used as a tool to support future facilitators’ preparation and facilitation of video-based programs.

### Drawing on classroom-based research and teacher learning research

**Fig. 1:**
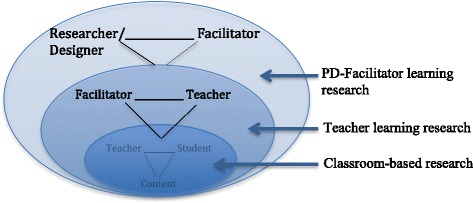
Layers of professional development design

As a field, we have experience in designing and studying case studies of PD aimed at teacher learning and improved student achievement in one particular setting. We have less experience designing and studying PD that “goes to scale.” Figure 1 depicts the layers of research and development on professional development that comprise efforts aimed at instructional improvement at scale.

The individuals on the right side of the ovals (regardless of which layer they are in) can be seen as learners; the individuals on the left side of the ovals can be seen as the teachers.^1^ In each layer, the “substance” of the teaching and learning activities is represented by the downward pointing vertex of the triangle. The innermost oval represents the classroom in which teachers work with students to learn the science or mathematics content (some refer to this as the instructional triangle; Cohen & Ball, 1999; 2000). In the middle layer, a PD facilitator helps a teacher to learn ways of helping students learn the content in the classroom. In the third layer, a researcher/program designer works with the PD facilitators to help them to learn how to help teachers to help students to learn. Because there is little systematic research focusing on the teaching and learning of PD facilitators, researchers and program designers often reach into “lower” levels of the diagram to appropriate ideas, tools, and resources to guide their work.

All of the studies in this issue draw on classroom-based research (inner layer) as well as teacher learning research (middle layer) to support their work in training facilitators. They draw on classroom-based research for two reasons. First, the target of PD is improved instructional practice and student learning. Consequently, designers and facilitators need to know and draw upon research on effective instructional practices in mathematics and science as they design their programs. Second, research conducted in classrooms that reveals key practices that support student learning can be useful when designing PD that will support teacher learning. Because research on teaching students is abundant, all four of the papers—at some point and to some degree of specification—reach out to frameworks, findings, and tools from classroom research. They also draw on teacher learning research to support the design of learning environments for novice facilitators who are learning how to support teacher development in PD.

Jacobs and colleagues reached into research on teaching to justify the assumptions that undergird their design principles and to build tools for supporting the PD facilitator. As they introduce their phase 2 work, they justify their focus on fidelity by reminding the reader of decades of curriculum research which established that teachers’ enactment of curriculum rarely completely matches the curriculum itself (with some changes being labeled as “lethal mutations”). Just like curriculum materials, PD programs, they argue, are susceptible to a wide range of adaptations. They also “borrowed” the tool of rehearsal from research on teacher preparation when designing tools for training of facilitators.

One might say that the entire edifice of STeLLA (Roth et al.) is built on careful attention to student thinking and teacher actions in the science classroom. A specific example of their building on classroom knowledge was their recognition of the need to incorporate teacher learning goals into their PD session agendas. Without a clear goal of what teachers were to “get out of” the session, discussions, they found, could drift in unproductive directions, similar to what they had seen when studying science lessons not guided by student learning goals.

The remaining papers also borrow from classroom research by focusing on the importance of supporting teachers’ learning toward carefully defined goals. For example, Tekkumru-Kisa and Stein stated, “when used by skilled facilitators, they [goals] can become the ‘north star’ by which to steer participants’ contributions toward a deeper understanding” (p. 8). Similarly, Borko and colleagues emphasize the role of goals in helping teacher leaders to recognize how much explicit attention they needed to devote to the planning of video-based discussions. The authors also emphasized that videos should be selected with a clear goal in mind. In addition, this group drew on Grossman and colleagues’ (2009) framework for the teaching of complex professional practice to pre-service teachers to guide their thinking about the design and facilitation of experiences for the professional development facilitators. Finally, both Tekkumru-Kisa and Stein, and Borko and colleagues adapted a tool based on classroom practice (Five Practices for Orchestrating Productive Discussions— Smith & Stein, 2011) for their professional development work with teachers.

Figure 1 also makes transparent the complexity of taking effective PD programs to scale. Often discussions about “going to scale” are framed by numbers, as in the number of teachers that will be “touched” and the number of personnel that are needed to be trained so that they, in turn, can train others (e.g., the train-the-trainers approach). Such discussions are often silent with respect to the substance of what must be learned at different layers of the system. Cast within a teaching and learning approach, Fig. 1 shows the complexity of the content to be learned, the amount of which rapidly increases as you move up the layers. For example, in the top layer, researchers/designers support facilitators to learn the substance in layer 1 (what effective math and science teaching and learning looks like in the classroom) and in layer 2 (how to work with teachers-as-learners to improve what they do in the classroom). This represents a steep learning curve for most researchers/designers as well as the facilitators. All of this suggests the importance of keeping a learning perspective on issues related to scale because it requires us to think carefully and conceptually about the requirements—many of which may be hidden from view—of rapidly expanding an effective PD program.

## Conclusions and looking forward

Our intention in creating this special issue is to facilitate and deepen the field’s thinking about video-based PD in mathematics and science and about what it takes to bring effective video-based PD programs to scale. The papers in this issue illustrate an important requirement of scaling up: making the work of PD facilitators visible such that researchers/designers can begin to more explicitly articulate their roles, how they learn to enact those roles, and how researchers/designers can support their learning. Looking forward, we can expect increasing pressure on both the science education and mathematics education communities to expand effective PD programs to larger and larger numbers of teachers. This suggests the need for more phase 2 and phase 3 research studies, especially those that use video as a central teaching tool.

These demands on PD research overlap with methodological improvements in education research. For example, design-based implementation research “is an emerging form of design research that supports the productive adaption of programs as they go to scale” (Penuel & Fishman, 2012; p. 282). It aims to include both the design of interventions and the ongoing improvement of the implementation of these interventions. As the field is now challenged to devise strategies for taking effective video-based PD programs beyond pockets of success and excellence to a larger scale, the DBIR approach can offer guidance, as illustrated in the paper by Borko and colleagues.

Our hope is that by providing concrete examples of the issues that confront researchers during the design and facilitation of video-based PD programs and doing so within the frame of Borko’s (2004) three phases of PD research, we have created a platform for looking forward as a field in this area of research. The final paper in this special issue is a commentary by Elizabeth van Es and Miriam Sherin that discusses the strengths and weaknesses of the papers as well as commenting on the extent to which the overall set of papers as a whole contributes to the ongoing dialogue about video-based PD and its scalability.

## Footnotes

1 Notice that one can be a teacher in one layer and a learner in another.

## References

[CR1] Ball DL, Cohen DK, Darling-Hammond L, Sykes G (1999). Developing practice, developing practitioners: toward a practice-based theory of professional education. Teaching as the learning profession.

[CR2] Banilower E, Smith PS, Weiss IR, Malzahn KA, Campbell KM, Weiss AM (2013). Report of the 2012 national survey of science and mathematics education.

[CR3] Banilower, E. R., Boyd, S. E., Pasley, J. D., & Weiss, I. R. (2006). Lessons from a decade of mathematics and science reform: a capstone report for the local systemic change through teacher enhancement initiative. Horizon Research*,* Inc*.*

[CR4] Bell, CA, Wilson, SM, Higgins, T, McCoach, DB. (2010). Measuring the effects of professional development on teacher knowledge: the case of developing mathematical ideas. *Journal for Research in Mathematics Education*, 479–512.

[CR5] Borko H (2004). Professional development and teacher learning: mapping the terrain. Educational Researcher.

[CR6] Borko H, Jacobs J, Eiteljorg E, Pittman ME (2008). Video as a tool for fostering productive discussions in mathematics professional development. Teaching and Teacher Education.

[CR7] Borko H, Jacobs J, Koellner K, Swackhamer LE (2015). Mathematics professional development: improving teaching using the problem-solving cycle and leadership preparation models.

[CR8] Borko, H., & Koellner, K. (2008). *Situativity: a theoretical lens for designing and studying programs of professional development*. Paper presented at the International Commission on Mathematical Instruction Symposium, Rome, March 2–5.

[CR9] Borko H, Koellner K, Jacobs J (2014). Examining novice teacher leaders’ facilitation of mathematics professional development. The Journal of Mathematical Behavior.

[CR10] Borko H, Koellner K, Jacobs J, Seago N (2011). Using video representations of teaching in practice-based professional development programs. ZDM.

[CR11] Brophy J (2004). Using video in teacher education.

[CR12] Brown AL (1992). Design experiments: theoretical and methodological challenges in creating complex interventions in classroom settings. The Journal of the Learning Sciences.

[CR13] Clark PG, Moore KC, Carlson MP (2008). Documenting the emergence of “speaking with meaning” as a socio-mathematical norm in professional learning community discourse. The Journal of Mathematical Behavior.

[CR14] Cohen, D. K., & Ball, D. L. (1999). Instruction, capacity, and improvement (CPRE Research Report No. RR-043). Philadelphia, PA: University of Pennsylvania, Consortium for Policy Research in Education.

[CR15] Cohen DK, Ball DL (2000). Instructional innovation: Reconsidering the story. Paper presented at the meeting of the American Educational Research Association.

[CR16] Coles, A. (2013). Using video for professional development: the role of the discussion facilitator. *Journal of Mathematics Teacher Education*, 1–20.

[CR17] Elliott R, Kazemi E, Lesseig K, Mumme J, Carroll C, Kelley-Petersen M (2009). Conceptualizing the work of leading mathematical tasks in professional development. Journal of Teacher Education.

[CR18] Even R (2008). Facing the challenge of educating educators to work with practicing mathematics teachers. The International Handbook of Mathematics Teacher Education.

[CR19] Garet MS, Porter AC, Desimone L, Birman BF, Yoon KS (2001). What makes professional development effective? Results from a national sample of teachers. American Educational Research Journal.

[CR20] Gaudin C, Chaliès S (2015). Video viewing in teacher education and professional development: a literature review. Educational Research Review.

[CR21] Goldsmith LT, Seago N, Sherin M, Jacobs V, Phillipp R (2011). Using classroom artifacts to focus teachers’ noticing. Mathematics teacher noticing: seeing through teachers’ eyes.

[CR22] Greeno JG, Sawyer RK (2006). Learning in activity. The Cambridge Handbook of the Learning Sciences.

[CR23] Greeno JG, Collins AM, Resnick LB (1996). Cognition and learning. Handbook of Educational Psychology.

[CR24] Grossman P, Compton C, Igra D, Ronfeldt M, Shahan E, Williamson P (2009). Teaching practice: a cross-professional perspective. Teachers College Record.

[CR25] Hawley, W. D., & Valli, L. (1999). The essentials of effective professional development. In L. Darling-Hammond & G. Sykes (Eds.), Teaching as the learning profession: Handbook of policy and practice (pp. 127–150). San Francisco: Jossey-Bass.

[CR26] Heller JI, Daehler KR, Wong N, Shinohara M, Maritrix LW (2012). Differential effects of three professional development models on teacher knowledge and student achievement in elementary science. Journal of Research in Science Teaching.

[CR27] Jackson K, Cobb P, Wilson J, Webster M, Dunlap C, Appelgate M (2015). Investigating the development of mathematics leaders’ capacity to support teachers’ learning on a large scale. ZDM.

[CR28] Koellner K, Jacobs J (2015). Distinguishing models of professional development: the case of an adaptive model’s impact on teachers’ knowledge, instruction, and student achievement. Journal of Teacher Education.

[CR29] Koellner K, Jacobs J, Borko H (2011). Mathematics professional development: critical features for developing leadership skills and building teachers' capacity. Mathematics Teacher Education and Development.

[CR30] Lesseig, K, Elliott, R, Kazemi, E, Kelley-Petersen, M, Campbell, M, Mumme, J, Carroll, C. (2016). Leader noticing of facilitation in videocases of mathematics professional development. *Journal of Mathematics Teacher Education*, 1–29.

[CR31] Loucks-Horsley S, Stiles KE, Mundry S, Love N, Hewson PW (2010). Designing professional development for teachers of science and mathematics.

[CR32] Marrongelle K, Sztajn P, Smith M (2013). Scaling up professional development in an era of common state standards. Journal of Teacher Education.

[CR33] Miller K, Zhou X, Goldman R, Pea R, Barron B, Derry SJ (2007). Learning from classroom video: what makes it compelling and what makes it hard. Video research in the learning sciences.

[CR34] National Academies of Sciences, Engineering, and Medicine (2015). *Science teachers learning: enhancing opportunities, creating supportive contexts*. Committee on Strengthening Science Education through a Teacher Learning Continuum. Board on Science Education and Teacher Advisory Council. Division of Behavioral and Social Science and Education.

[CR35] Penuel WR, Fishman BJ (2012). Large-scale science education intervention research we can use. Journal of Research in Science Teaching.

[CR36] Penuel WR, Fishman BJ, Yamaguchi R, Gallagher LP (2007). What makes professional development effective? Strategies that foster curriculum implementation. American Educational Research Journal.

[CR37] Penuel WR, Gallagher LP, Moorthy S (2011). Preparing teachers to design sequences of instruction in earth systems science: a comparison of three professional development programs. American Educational Research Journal.

[CR38] Putnam RT, Borko H (2000). What do new views of knowledge and thinking have to say about research on teacher learning?. Educational Researcher.

[CR39] Reiser, B. J. (2013). *What professional development strategies are needed for successful implementation of the Next Generation Science Standards?* White paper presented to the Invitational Research Symposium on Science Assessment. The Center for K-12 Assessment & Performance Management at Educational Testing Service (ETS).

[CR40] Roth KJ, Garnier HE, Chen C, Lemmens M, Schwille K, Wickler NIZ (2011). Videobased lesson analysis: effective science PD for teacher and student learning. Journal of Research in Science Teaching.

[CR41] Schifter D, Lester JB (2005). Active facilitation: What do facilitators need to know and how might they learn it?. Journal of Mathematics and Science: Collaborative Explorations.

[CR42] Sherin MG, Brophy J (2004). New perspectives on the role of video in teacher education. Advances in research on teaching: Using video in teacher education (Vol. 10).

[CR43] Sherin MG, Han SY (2004). Teacher learning in the context of a video club. Teaching and Teacher Education.

[CR44] Smith M, Stein MK (2011). Five practices for orchestrating productive mathematics discussions.

[CR45] Stein MK, Smith MS, Silver EA (1999). The development of professional developers: Learning to assist teachers in new settings in new ways. Harvard Educational Review.

[CR46] Tekkumru-Kisa, M. & Stein, M. K. (2015a). Learning to see teaching in new ways: A foundation for maintaining cognitive demand. American Educational Research Journal, 52(1), 105–136.

[CR47] van Es EA, Sherin MG (2010). The influence of video clubs on teachers’ thinking and practice. Journal of Mathematics Teacher Education.

[CR48] van Es EA, Tunney J, Goldsmith L, Seago N (2014). A framework for the facilitation of teachers’ analysis of video. Journal of Teacher Education.

[CR49] van Es E, Tunney J, Seago N, Goldsmith LT, Calandra B, Rich PJ (2015). Facilitation practices for supporting teacher learning with video. Digital video for teacher education: research and practice.

[CR50] Wilson SM (2013). Professional development for science teachers. Science.

